# Association between occupational noise-induced hearing loss and cognitive function: a multimodal cross-sectional study

**DOI:** 10.3389/fpubh.2026.1789178

**Published:** 2026-04-10

**Authors:** Qi Jin, Yangfu Bian, Cailing Zhou

**Affiliations:** Department of Occupational Disease, Hangzhou Hospital for the Prevention and Treatment of Occupational Disease, Hangzhou, China

**Keywords:** auditory brainstem response, cognitive impairment, hearing loss, hypertension, occupational noise exposure, smoking

## Abstract

**Objective:**

To investigate the association between hearing loss and cognitive function in noise-exposed workers, to evaluate the predictive value of multimodal audiological and neurophysiological indicators, and to analyze the influence of covariates, including smoking, alcohol consumption, hypertension, and diabetes.

**Methods:**

In this cross-sectional study, 170 noise-exposed workers with at least 6 months of exposure were enrolled from 2023 to 2025 using cluster sampling. Participants underwent pure-tone audiometry, tympanometry, otoacoustic emissions (OAEs), auditory brainstem response (ABR), and the Montreal Cognitive Assessment (MoCA). Data on demographics, smoking, alcohol consumption, hypertension, and diabetes were collected. Pearson correlation, multiple linear regression, hierarchical regression, and bootstrap mediation analysis were performed.

**Results:**

The study included 170 participants, of whom 97.1% were male. The mean age was 46.9 ± 9.8 years, and the mean duration of noise exposure was 6.9 ± 5.7 years. The mean high-frequency hearing threshold was 54.9 ± 10.2 dB, and the mean MoCA score was 26.2 ± 2.1. Hearing thresholds and ABR wave V latency were both significantly negatively correlated with MoCA scores (*p* < 0.05). The absolute correlation coefficients ranged from 0.2 to 0.3, indicating weak to moderate correlations. Multiple regression analysis showed that age, exposure duration, and high-frequency hearing threshold were independent predictors of MoCA score (*p* < 0.05). This association remained stable after adjustment for smoking, alcohol consumption, hypertension, and diabetes. Mediation analysis revealed that hearing loss played a significant mediating role in the negative effect of occupational hazard exposure duration on cognitive function. Specifically, 47.6% of the total effect of exposure duration on cognitive function was mediated by high-frequency hearing loss. The observed correlation coefficients were modest (e.g., *r* ≈ −0.3), suggesting a small to moderate clinical effect size. Hypertension was also independently associated with lower MoCA scores (*β* = −0.120, *p* = 0.048).

**Conclusion:**

In this predominantly male cohort of noise-exposed workers, hearing loss was significantly associated with cognitive decline. This association was independent of age, exposure duration, and other health risk factors, including smoking, alcohol consumption, hypertension, and diabetes. These findings suggest a potential link between occupational hearing loss and cognitive function. However, because of the limitations of the cross-sectional design, causal relationships cannot be inferred. A multimodal assessment integrating audiological, neurophysiological, and health risk factors may provide a more systematic and objective reference for early identification, risk warning, and the development of comprehensive occupational health intervention strategies for cognitive decline in occupational populations.

## 1 Introduction

Prolonged occupational noise exposure is a major risk factor for sensorineural hearing loss, namely occupational noise-induced hearing loss (ONIHL) (1). Traditional research has focused primarily on damage to the auditory system. However, increasing evidence suggests that the adverse effects of noise exposure extend beyond the cochlea and may negatively affect the central nervous system through multiple pathways, including auditory deprivation, increased cognitive load, stress responses, and even direct central nervous system injury (2, 3). Notably, these effects are closely associated with cognitive decline (4, 5). Epidemiological studies have further confirmed that occupational noise exposure is a potential risk factor for mild cognitive impairment and even dementia (6, 7).

The impact of noise exposure on cognitive function may involve multiple domains, including attention, memory, learning, and working ability (8, 9). Existing studies examining the relationships among noise, hearing, and cognition have often used age, years of service, and noise exposure as the primary control variables. However, insufficient attention has been given to lifestyle and metabolic factors that affect cognition and vascular health, such as smoking, alcohol consumption, hypertension, and diabetes (10, 11). These factors are themselves risk factors for cognitive decline and microvascular disease of the auditory system. Therefore, they may serve as important confounders or effect modifiers and may interfere with accurate assessment of the independent effect of noise exposure on cognitive decline (12).

Domestic studies have predominantly relied on pure-tone audiometry (PTA) for hearing assessment and have employed simplified scales for cognitive screening. As a result, systematic multimodal research addressing the continuous pathway of “peripheral hearing-central auditory processing-higher cognitive functions” remains limited (13). Auditory brainstem response (ABR), as an objective assessment tool, effectively reflects neural conduction from the peripheral auditory organs to the brainstem and is particularly useful for evaluating the physiological status of the auditory brainstem. It therefore provides critical neurophysiological evidence for in-depth investigation of the relationship between hearing and cognition (14, 15).

This study conducted a cross-sectional investigation of occupational noise-exposed individuals in a specific region from 2023 to 2025. Comprehensive multimodal assessment methods were applied, including pure-tone audiometry, acoustic immittance testing (AIT), otoacoustic emissions (OAEs), brainstem auditory evoked potentials (BAAEPs), and the Montreal Cognitive Assessment (MoCA). Covariates such as smoking, alcohol consumption, hypertension, and diabetes were also systematically incorporated. The objectives were to clarify the association between hearing loss and cognitive function in noise-exposed workers, to explore the potential role of central auditory processing in this relationship, and to evaluate the independent influence of common health risk factors on this association. The findings are expected to provide empirical evidence for the development of a more precise occupational health risk assessment system and comprehensive intervention strategies.

## 2 Study subjects

### 2.1 Sampling method

Cluster sampling was used to select workers exposed to noise intensity ≥ 85 dB(A) from multiple industrial enterprises in a specific region during 2023–2025. Enterprises were selected according to their scale, industry representativeness, and concentration of noise-exposed workers. All workers in the selected enterprises who met the inclusion and exclusion criteria were invited to participate.

### 2.2 Inclusion criteria

① Age ≥ 18 years; ② workers exposed to noise intensity ≥ 85 dB(A) with a cumulative exposure duration ≥ 6 months; ③ individuals diagnosed with sensorineural hearing loss; ④ provision of informed consent and cooperation with all examinations.

### 2.3 Exclusion criteria

① Non-noise-related ear disorders, such as otitis media, a history of otologic surgery, or congenital deafness; ② a history of confirmed central nervous system diseases, severe mental disorders, or dementia; ③ a history of alcohol or drug abuse. Educational level and history of psychotropic drug use were not included as exclusion criteria, nor were they matched or adjusted for. This represents a limitation of the study. All participants provided written informed consent. This study was approved by the Medical Ethics Committee (approval no. HZF-IRB-2022-001).

## 3 Research methods

### 3.1 Auditory and neurophysiological examinations

#### 3.1.1 Pure-tone hearing threshold testing

Pure-tone hearing thresholds were measured in an anechoic chamber under standard conditions (ambient noise ≤ 30 dB) using an Otometrics Itera audiometer (Denmark) with matched TDH-39 earphones. The test stimulus was a pure tone, with a maximum output intensity of 120 dB HL, covering frequencies of 0.5, 1.0, 2.0, 3.0, 4.0, and 6.0 kHz. The procedure strictly followed *Acoustics—Methods for Hearing Measurement—Part 1: Pure-tone Air Conduction and Bone Conduction Testing* (GB/T 16296.1-2018). Hearing thresholds were determined using the ascending method, in which sound stimuli were repeatedly presented according to the “down 10 dB, up 5 dB” principle. The threshold was defined as the lowest intensity at which responses were obtained in 2 of 3 ascending trials at the same frequency.

#### 3.1.2 Acoustic impedance and otoacoustic emission (OAE)

The examination was performed in a soundproof room with a background noise level ≤ 30 dB(A) using the Titan automated otoacoustic emission tester (Interacoustics, Denmark). The instrument parameters were set as follows: the ratio of the higher frequency (f₂) to the lower frequency (f₁) of the primary tones (f₂/f₁) was 1.22; the stimulus intensities were L₁ = 65 dB SPL and L₂ = 55 dB SPL; and the frequency range covered 0.5, 1.0, 2.0, 3.0, 4.0, and 6.0 kHz. Acoustic impedance, ipsilateral acoustic reflex threshold, DPOAE amplitude at each frequency, signal-to-noise ratio (SNR), and pass rates were automatically recorded and exported by the device. The DPOAE pass rate was calculated automatically by the device. A response was considered present when the signal amplitude was > − 5.0 dB and at least 6.0 dB above the background noise; this criterion was preset by the device.

#### 3.1.3 Brainstem auditory evoked potential (ABR)

The ABR test was conducted in an electromagnetic shielding room (ambient noise ≤ 30 dB) using the Eclipse EP25 auditory testing platform (Interacoustics, Denmark), with a maximum stimulus intensity of 100 dB nHL. EarTone ABR earphones (Otometrics, Denmark) were used. For electrode placement, the recording electrode was placed below the frontal hairline, the reference electrodes were positioned at both mastoids, and the ground electrode was placed at the nasal root. Electrode impedance was required to be ≤ 5 kΩ, and the artifact rejection criterion was ± 40 μV. The stimulus used was CE-Chirp, with a time window of 20 ms and 2000 sweeps, administered bilaterally. The stimulus intensity started at 80 dB nHL and was adjusted in 5 dB steps, with each intensity tested at least twice. The minimum stimulus intensity that reliably elicited a repeatable wave V was defined as the ABR response threshold. To avoid false-positive results, EEG stability and noise reduction during signal averaging were closely monitored. The test was performed by an experienced audiologist who was not blinded to the participant exposure history, which may have introduced measurement bias.

### 3.2 Cognitive function assessment

The Chinese version of the Montreal Cognitive Assessment (MoCA) was used for evaluation. The total score was 30, and a score < 26 indicated cognitive impairment. Scores were also recorded for seven cognitive subdomains: visuospatial/executive function, naming, attention, language, abstraction, delayed recall, and orientation.

### 3.3 General health and lifestyle survey

Structured questionnaires were administered through face-to-face interviews: ① Smoking: Participants were classified into three groups according to smoking frequency: non-smokers (never smoked or had quit smoking for ≥ 6 months), occasional smokers (non-daily smokers), and frequent smokers (daily smokers). ② Alcohol consumption: Participants were classified into three groups according to drinking frequency: non-drinkers (drinking fewer than once per week during the past 12 months), occasional drinkers (drinking 1–3 times per week during the past 12 months), and frequent drinkers (drinking ≥ 4 times per week during the past 12 months). ③ Hypertension: Hypertension was confirmed by occupational health records, on-site blood pressure measurement (≥ 140/90 mmHg), or a prior clinical diagnosis. ④ Diabetes mellitus: Diabetes mellitus was defined as fasting blood glucose ≥ 7.0 mmol·L^−1^, glycated hemoglobin (HbA1c) ≥ 6.5%, or a previous clinical diagnosis.

### 3.4 Statistical analysis

Statistical analysis was performed using SPSS 26.0. Continuous variables were expressed as mean ± standard deviation (x̄ ± s), and categorical variables were presented as *n* (%).

#### 3.4.1 Correlation analysis

Pearson correlation analysis was used to examine the correlations of hearing indicators, ABR indicators, and covariates with the total MoCA score. ABR wave V latency was included in the mediation model as an indicator of central auditory conduction function.

#### 3.4.2 Regression analysis

Multiple linear regression analysis (enter method) was used to identify independent factors associated with the total MoCA score. To further evaluate the independent contributions of health risk factors, hierarchical regression analysis was performed. Model 1 (baseline model) included age, sex, exposure duration, smoking, alcohol consumption, and hypertension. Model 2 added the binaural high-frequency average hearing threshold to Model 1. Model 3 added the left and right high-frequency average hearing thresholds separately to Model 1. Model 4 (full model) simultaneously included the binaural and unilateral hearing variables.

#### 3.4.3 Mediation analysis

The PROCESS macro (Model 4) was used to examine the multiple mediating pathways through which exposure duration affected cognitive function, with binaural high-frequency average hearing threshold, ABR wave V latency, smoking frequency, and hypertension included as mediators. Bootstrap resampling with 5,000 iterations was performed to estimate the 95% confidence intervals (CIs) for the indirect effects. An effect was considered statistically significant when the 95% CI did not include zero. A two-tailed *p* < 0.05 was considered statistically significant.

## 4 Results

### 4.1 Basic characteristics and key variables of the study subjects

A total of 170 individuals with occupational noise exposure were enrolled in this study, including 165 males (97.1%) and 5 females (2.9%). The mean age was 46.9 ± 9.8 years (range, 23–68 years), and most participants were middle-aged, with 70.6% aged 41–60 years. The mean duration of occupational noise exposure was 6.9 ± 5.7 years. Among the participants, 82 (48.2%) had an exposure duration of ≤ 5 years, 51 (30.0%) had 6–10 years, and 37 (21.8%) had > 10 years. Regarding health risk factors, 62.4% of the participants were non-smokers, 55.3% were non-drinkers, 25.3% had hypertension, and 0.6% had diabetes. Regarding preventive measures, 17.6% (30 participants) used hearing protection earplugs, whereas most participants (82.4%, 140 participants) did not use this preventive measure (Table 1). The distribution of primary occupations among the participants is shown in Table 2.

**Table 1:** Basic characteristics of study subjects (*N* = 170).

Feature	Category/indicator	Number of people (*n*)	Constituent ratio (%)	Mean ± standard deviation/range
Sex	The male sex	165	97.1	-
	Femininity	5	2.9	-
Age (years)	Ensemble	170	100	46.9 ± 9.8(23–68)
	≤40 years of age	36	21.2	-
	41–50 years old	68	40.0	-
	51–60 years old	52	30.6	-
	>60 years old	14	8.2	-
Years of service in the job	Ensemble	170	100	6.9 ± 5.7(0.5–30)
	≤5 years	82	48.2	-
	6–10 years	51	30.0	-
	>10 years	37	21.8	-
Smoking habit	Smoking	106	62.4	-
	Occasional smoking	34	20.0	-
	Regular smoking	30	17.6	-
Alcohol consumption habits	Do not drink alcohol.	94	55.3	-
	Occasional drinking	56	32.9	-
	Regular alcohol consumption	20	11.8	-
History of chronic diseases	Hypertension	43	25.3	-
	Diabetes mellitus	1	0.6	-
Use of hearing protection earplugs	Yes	30	17.6	
	No	140	82.4	

**Table 2:** Distribution of occupations.

Type of work in production	Number of people	Percentage	Average years of service
Electro-welding/welding technician	28	16.5%	8.2 ± 6.1
Grinder	25	14.7%	7.8 ± 5.9
Stamping machine operator	12	7.1%	6.5 ± 4.7
Researcher	10	5.9%	9.1 ± 7.2
Assemblers	9	5.3%	7.3 ± 5.4
Control room operator	8	4.7%	5.2 ± 4.1
Injection molding technician	7	4.1%	4.8 ± 3.9
Cutting machine	6	3.5%	6.7 ± 5.3
Packer	5	2.9%	10.2 ± 8.4
Other trades	60	35.3%	6.1 ± 5.0

Audiometric assessment showed that the bilateral high-frequency average hearing threshold was 54.9 ± 10.2 dB. According to WHO criteria, hearing impairment was predominantly moderate (45.9%) or moderately severe (29.4%). Notably, all participants failed the otoacoustic emissions test in both ears (100%). Acoustic immittance testing showed abnormal tympanograms in 61.2% of the participants. The high-frequency average hearing threshold was 54.0 ± 10.5 dB in the left ear and 55.8 ± 10.3 dB in the right ear. Paired *t*-test showed a statistically significant difference between the two ears (*t* = 2.13, *p* = 0.035) (Table 3). Cognitive assessment showed a mean MoCA score of 26.2 ± 2.1. Among the participants, 112 (65.9%) had normal cognitive function (≥ 26 points), whereas 58 (34.1%) had mild cognitive impairment (19–25 points). The subdomain scores are presented in Table 4.

**Table 3:** Results of auditory examination.

Inspection item	Detection result (mean ± SD/range)
Binaural high-frequency average hearing threshold (dB)	**54.9 ± 10.2 (35–100)**
Degree of hearing loss (WHO criteria)	
Mild (26–40 dB)	18 (10.6%)
Moderate (41–55 dB)	78 (45.9%)
Moderate to severe (56–70 dB)	50 (29.4%)
Severe (71–90 dB)	20 (11.8%)
Extremely loud (≥91 dB)	4 (2.4%)
Auditory brainstem response threshold (dBnHL)	Right ear: 52.3 ± 10.5 (35–75)
	Left ear: 53.1 ± 11.2 (35–80)
Otoacoustic emission testing	Both ears failed (100%)
acoustic impedance audiometry	Abnormal tympanogram ratio: 61.2%
Mean high frequency auditory threshold of left and right ears	Left ear: 54.0 ± 10.5 dB
	Right ear: 55.8 ± 10.3 dB
	Difference: 1.8 dB
	Paired t-test: t = 2.13, P = 0.035

The comparison of high-frequency average hearing thresholds between the left and right ears was performed using paired t-tests; the high-frequency average hearing thresholds were the mean values at 3000, 4000, and 6,000 Hz. The bolded value 54.9 ± 10.2 (35–100) is the binaural high‑frequency average hearing threshold (mean ± SD, range). It was significantly correlated with cognitive function (*p* < 0.01) and used as the main hearing loss indicator in this study.

**Table 4:** Cognitive function assessment (MoCA) in study subjects.

Evaluate projects	Full marks	Score (mean ± SD)	Scoring average (%)
MoCA total points	30	26.2 ± 2.1	87.3
Visual space/executive function	5	4.4 ± 0.6	88.0
Nominate	3	2.9 ± 0.3	96.7
Pay attention to	6	5.3 ± 0.8	88.3
Language	3	2.8 ± 0.4	93.3
Abstract	2	1.9 ± 0.3	95.0
Delayed recall	5	4.5 ± 0.9	90.0
Directive force	6	5.3 ± 0.7	88.3
Normal cognition (≥26 points)		112(65.9%)	
Mild cognitive impairment (19–25 points)		58(34.1%)	

### 4.2 Correlation analysis between hearing, neurophysiological indicators, and cognitive function

The results showed that all auditory parameters, including the left and right high-frequency average thresholds and frequency-specific thresholds, were significantly negatively correlated with the total MoCA score (all *p* < 0.05). Comparison between the two ears showed that the correlation between the left high-frequency average threshold and the total MoCA score (*r* = −0.28) was slightly stronger than that for the right ear (*r* = −0.25). Among the ABR-related parameters, the I–III interval, III–V interval, and wave V latency were significantly negatively correlated with the total MoCA score (all *p* < 0.001), whereas wave V amplitude and the V/I wave amplitude ratio showed weak positive correlations (*p* < 0.05) (Table 5). The absolute values of these correlation coefficients ranged from 0.2 to 0.3, indicating weak to moderate correlations. The bilateral high-frequency average thresholds were significantly negatively correlated with the total MoCA score (*r* = −0.31, *p* < 0.01) and significantly positively correlated with age (*r* = 0.42, *p* < 0.01) and work tenure (*r* = 0.38, *p* < 0.01). Hypertension was also significantly negatively correlated with the total MoCA score (*r* = −0.24, *p* < 0.01) (Table 6).

**Table 5:** Correlation analysis between auditory and ABR indices and MoCA total score (including left and right ear high-frequency average threshold).

Indicator category	Name of index	Left ear r (*p*-value)	Right ear (*p*-value)
Hearing indicators	**High frequency mean threshold**	**−0.28 (*p* < 0.001)**	**−0.25 (*p* = 0.001)**
	Hearing threshold weighting	−0.28 (*p* < 0.001)	−0.26 (*p* < 0.001)
	3,000 Hz hearing threshold	−0.25 (*p* = 0.001)	−0.24 (*p* = 0.002)
	4,000 Hz hearing threshold	−0.27 (*p* < 0.001)	−0.25 (*p* = 0.001)
	6,000 Hz hearing threshold	−0.22 (*p* = 0.005)	−0.23 (*p* = 0.003)
ABR metric	I-III interval	−0.31 (*p* < 0.001)	−0.29 (*p* < 0.001)
	III-V interval	−0.27 (*p* < 0.001)	−0.25 (*p* = 0.001)
	v-wave latency	−0.33 (*p* < 0.001)	−0.31 (*p* < 0.001)
	V amplitude	0.18 (*p* = 0.022)	0.16 (*p* = 0.042)
	V/I wave amplitude ratio	0.20 (*p* = 0.010)	0.18 (*p* = 0.023)

r: Pearson correlation coefficient, positive values indicate positive correlation, negative values indicate negative correlation. *p*-value: significance level, *p* < 0.05 indicates significant correlation. For the high frequency mean threshold row, bolded r = –0.28 (left ear, *p* < 0.001) and –0.25 (right ear, *p* = 0.001) indicate significant negative correlations with MoCA total score.

**Table 6:** Pearson correlation coefficients among variables.

variable	Binaural high-frequency average hearing threshold	MoCA total points	Age	Noise exposure duration	Frequency of smoking	Frequency of alcohol consumption	Hypertension
Binaural high-frequency average hearing threshold	1.00	−0.31**	0.42**	0.38**	0.18*	0.15*	0.12
MoCA total points	−0.31**	1.00	−0.29**	−0.26**	−0.09	−0.11	−0.24**
Age	0.42**	−0.29**	1.00	0.45**	0.08	0.10	0.21**
Noise exposure duration	0.38**	−0.26**	0.45**	1.00	0.06	0.07	0.18*
Frequency of smoking	0.18*	−0.09	0.08	0.06	1.00	0.32**	0.13
Frequency of alcohol consumption	0.15*	−0.11	0.10	0.07	0.32**	1.00	0.09
Hypertension	0.12	−0.24**	0.21**	0.18*	0.13	0.09	1.00

* indicates *p* < 0.05, ** indicates *p* < 0.01.

### 4.3 Multivariate linear regression analysis of cognitive function

This study constructed multiple linear regression models to investigate the independent factors associated with cognitive function (Table 7). Model 1 (baseline model) included age, sex, exposure duration, smoking, alcohol consumption, and hypertension, and explained 19% of the variance in the total MoCA score (*R*^2^ = 0.19). Among these variables, age (*β* = −0.11, *p* < 0.01), exposure duration (*β* = −0.15, *p* < 0.05), and hypertension (β = −0.45, *p* < 0.05) showed significant negative predictive effects on the total MoCA score. Model 2 added the bilateral high-frequency average hearing threshold to the baseline model, resulting in an increase in *R*^2^ of 0.16 (Δ*R*^2^ = 0.16, *p* < 0.001). In this model, the bilateral high-frequency average hearing threshold showed a significant negative predictive effect on the total MoCA score (*β* = −0.15, *p* < 0.001). Model 3 added the left and right high-frequency average hearing thresholds to Model 1. The left high-frequency average hearing threshold showed a marginally significant negative predictive effect on the total MoCA score (*β* = −0.08, *p* = 0.066), whereas the effect of the right ear was not significant (β = −0.07, *p* = 0.101). Model 4 (full model) included both bilateral and unilateral hearing variables. The bilateral high-frequency average hearing threshold remained a significant negative predictor (*β* = −0.10, *p* < 0.05), whereas the unilateral variables were no longer significant.

**Table 7:** Results of multivariate linear regression analysis with multiple models for factors influencing MoCA total score (*N* = 170).

Predictive variable	Model 1: basic variables	Model 2: +binaural high-frequency average threshold	Model 3: + high-frequency hearing threshold in both ears	Model 4: full model
Constant term	29.78 (1.25)***	29.45 (1.23)***	29.52 (1.24)***	29.40 (1.24)***
Demographic variables				
Age (years)	−0.11 (0.03)**	−0.08 (0.03)*	−0.08 (0.03)*	−0.08 (0.03)*
Gender (Male)	−0.15 (0.46)	−0.18 (0.45)	−0.19 (0.45)	−0.20 (0.45)
Health behavior variables				
Years of service in the job	−0.15 (0.06)*	−0.12 (0.06)*	−0.12 (0.06)*	−0.12 (0.06)*
Frequency of smoking	0.07 (0.08)	0.05 (0.08)	0.05 (0.08)	0.05 (0.08)
frequency of alcohol consumption	−0.09 (0.07)	−0.07 (0.07)	−0.07 (0.07)	−0.07 (0.07)
Hypertension (present)	−0.45 (0.21)*	−0.52 (0.21)*	−0.51 (0.21)*	−0.51 (0.21)*
Hearing variables				
Binaural high-frequency average hearing threshold (dB)	—	−0.15 (0.04)***	—	−0.10 (0.05)*
Average high-frequency hearing threshold (dB) of the left ear	—	—	−0.08 (0.04)†	−0.05 (0.05)
Average high-frequency hearing threshold (dB) of the right ear	—	—	−0.07 (0.04)	−0.05 (0.05)
Model statistic				
R^2^	0.19	0.35	0.34	0.35
Adjust R^2^	0.16	0.32	0.31	0.31
R^2^ change	—	0.16***	0.15***	0.16***
F price	7.65***	12.87***	11.23***	10.85***
Maximum VIF	1.28	1.52	1.63	2.45
Average VIF	1.15	1.25	1.32	1.65

The values in the table represent β coefficients (standard errors), with *p < 0.05, **p < 0.01, ***p < 0.001, and †*p* < 0.10 (marginally significant). The average high-frequency auditory threshold for both ears = (3,000 Hz + 4,000 Hz + 6,000 Hz)/3. All models exhibit a VIF <5, indicating no severe multicollinearity.

To clarify the pathway through which exposure duration affected cognitive function, bootstrap mediation analysis was performed. The results showed a significant negative total effect of occupational noise exposure duration on the total MoCA score (*β* = −0.210, 95% CI: −0.330 to −0.090). After adjustment for the mediators, the direct effect was no longer statistically significant (β = −0.060, 95% CI: −0.190 to 0.070). Among the indirect pathways, the effect mediated by the bilateral high-frequency average hearing threshold was significant (β = −0.100, 95% CI: −0.210 to −0.010), accounting for 47.6% of the total effect. These findings suggest that bilateral high-frequency hearing loss played a significant mediating role in the association between exposure duration and cognitive function in the present model.

This study found that the degree of hearing loss in individuals exposed to occupational noise was significantly negatively correlated with cognitive function, and this association was independent of age, years of service, and common health risk factors. This finding is consistent with previous studies (16). Noise exposure is not only a damaging factor for the peripheral auditory system, but may also be an independent risk factor for cognitive decline. The underlying mechanisms may involve auditory deprivation, increased cognitive load, and indirect injury to the central nervous system.

Mediation analysis further showed that hearing loss played a significant mediating role in the relationship between noise-exposed work duration and cognitive function, accounting for 47.6% of the total effect. This finding suggests that hearing impairment is a critical pathway through which noise affects cognitive function. This result is consistent with the cognitive load hypothesis, which proposes that hearing loss forces the brain to continuously allocate additional cognitive resources to auditory processing, thereby reducing the resources available for higher cognitive functions such as memory encoding and executive control. In this study, delayed recall and cognitive subdomains such as visuospatial and executive function showed relatively strong associations with hearing loss (Tables 4, 5), providing indirect support for this hypothesis (17–19). In addition, previous studies have shown that cognitive reserve accumulated through long-term music training may alleviate speech perception difficulties in noisy environments by optimizing neural resource allocation (20), further supporting the regulatory role of cognitive resources in the auditory-cognitive association.

Regarding the pattern of hearing loss, this study focused on the high-frequency hearing thresholds at 3, 4, and 6 kHz, which are the typical frequencies affected by occupational noise-induced hearing loss and the core indicators for diagnosis according to the national occupational health standard of China, *Technical Specifications for Occupational Health Surveillance* (GBZ 188–2025). From the perspective of occupational health management, exposure duration may serve as a practical marker of cumulative noise exposure. In this study, exposure duration showed a significant positive correlation with the bilateral high-frequency average hearing threshold (*r* = 0.38, *p* < 0.01), and hearing loss was significantly greater among individuals with > 10 years of exposure than among those with shorter exposure durations. This trend is consistent with previous studies.

Notably, these findings are consistent with those reported by Aliyeva and Sari in a Turkish occupational population. Their study also showed that noise-exposed workers had significantly elevated hearing thresholds at high frequencies, particularly at 4 and 6 kHz. They also reported elevation at 8 kHz, which was not tested in the present study. In addition, hearing loss was significantly greater among individuals with > 5 years of exposure than among those with ≤ 5 years. Importantly, tinnitus was identified as the primary predictor of noise-induced hearing loss (OR = 2.126, 95% CI: 0.99–3.70). Although tinnitus data were not systematically collected in the present study, these findings suggest that tinnitus may represent an early clinical warning sign of noise-induced auditory damage and therefore deserves attention in occupational health surveillance (21). Furthermore, the low rate of hearing protection use observed in this study, with 82.4% of workers not using hearing protection, underscores the need to strengthen hearing protection training and intervention in occupational populations.

In this study, ABR wave V latency showed a significant negative correlation with the total MoCA score (left ear: *r* = −0.33; right ear: *r* = −0.31; *p* < 0.001; Table 5) and was independently associated with the bilateral high-frequency hearing threshold. This finding is consistent with the hypothesis that noise exposure may affect neural conduction at the brainstem level. However, because of the cross-sectional design and the lack of neuroimaging evidence, direct injury to the central auditory pathway caused by noise exposure cannot be inferred (22–25). Therefore, ABR findings should be interpreted with caution.

Although the independent predictive roles of the left and right high-frequency hearing thresholds in the multiple regression analysis were not statistically significant (left ear: *p* = 0.066; right ear: *p* = 0.101; Table 7), the correlation coefficient between the left ear and the total MoCA score was slightly greater than that for the right ear (*r* = −0.28 vs. −0.25; Table 5). This finding suggests that left-ear hearing loss may show a slightly stronger association with cognitive function and warrants further investigation. According to the theory of auditory processing lateralization, left-ear input is primarily projected to the right hemisphere, which may exert a more direct influence on cognitive functions such as spatial perception, nonverbal information processing, and music-related memory. Neuroimaging studies have also shown that regions such as the left parahippocampal cortex play an important role in linking central auditory processing and cognitive function, providing a possible direction for understanding the neural mechanisms underlying unilateral hearing impairment. Future studies may perform stratified analyses of specific cognitive subdomains, such as visuospatial function and delayed recall, to further examine this hypothesis.

Among the covariates, hypertension was identified as an independent negative predictor of cognitive function (standardized *β* ≈ −0.12, *p* = 0.048; Table 7, Model 2), suggesting that hypertension may aggravate inner ear injury through microvascular complications and thereby indirectly increase cognitive load (26). Mediation analysis showed that although the indirect effect of the hypertension pathway was not statistically significant, accounting for 4.8% of the total effect (*p* > 0.05; Table 8), its direction was consistent with the proposed noise-cardiovascular-cochlea-cognition cascade hypothesis (27). Smoking, alcohol consumption, and diabetes did not show significant independent or mediating effects in this study. This may be attributable to the limited sample size, the very low prevalence of diabetes (only 1 case), and the relatively coarse classification of smoking and alcohol consumption. Further validation in larger samples is needed.

**Table 8:** Mediating effect analysis of years of exposure to hazard on MoCA total score.

Effect path	Effect size	Boot standard error	BootCI lower limit	BootCI superior limit	Relative effect ratio
Gross effect	−0.210	0.060	−0.330	−0.090	100%
Direct effect (exposure duration → MoCA)	−0.060	0.065	−0.190	0.070	28.6%
Indirect effects (exposure duration → hearing threshold → MoCA)	−0.100	0.055	−0.210	−0.010	47.6%
Indirect effects (exposure duration → ABR wave V latency → MoCA)	−0.030	0.025	−0.080	0.010	14.2%
Indirect effects (noise exposure duration → smoking → MoCA)	−0.010	0.020	−0.050	0.030	4.8%
Indirect effects (exposure duration → hypertension → MoCA)	−0.010	0.025	−0.060	0.040	4.8%

Bootstrap sample size = 5,000; CI refers to confidence interval; the sum of path effect proportions equals 100%.

Approximately 47.6% of the effect of noise exposure duration on cognitive function was mediated through high-frequency hearing loss (indirect effect: −0.100, 95% CI: −0.210 to −0.010). This finding further supports the central role of hearing protection in maintaining cognitive health in occupational populations. In contrast, the indirect effect through the ABR pathway did not reach statistical significance (−0.030, 95% CI: −0.080 to 0.010), suggesting that central auditory conduction may not be the primary pathway through which noise affects cognition in this study population, or that more sensitive neurophysiological indicators may be required.

The 100% failure rate of distortion product otoacoustic emissions (DPOAE) in this study warrants attention. This finding may be related to the inclusion of individuals with confirmed hearing loss, but it may also suggest the presence of hidden hearing loss, that is, synaptic lesions of inner hair cells that cannot be detected by conventional pure-tone audiometry (28). Future studies may use more sensitive techniques, such as electrically evoked compound action potentials (ECAP), to further investigate this mechanism (29).

Based on these findings, implementation of systematic interventions from the perspective of occupational health management is of substantial importance. It is recommended that cognitive function screening, such as MoCA, be incorporated into occupational health surveillance for high-risk noise-exposed populations. Objective neurophysiological examinations such as ABR may serve as a supplement to pure-tone audiometry for identifying individuals at high risk of central auditory processing disorder (30). For individuals with normal pure-tone hearing thresholds but abnormal ABR findings or failed OAE, early attention and intervention should be provided.

This study has several limitations. First, the cross-sectional design precludes causal inference, and prospective cohort studies are needed to verify the causal direction suggested by the present findings. Second, the relatively limited sample size and marked sex imbalance (97.1% male) limit the generalizability of the findings to female occupational populations (31). In addition, the lack of control for educational level may have introduced confounding in the cognitive assessment, and this issue should be addressed in future studies. Third, noise exposure assessment relied solely on exposure duration as a proxy indicator and did not include individualized noise dose data, which may have introduced measurement bias into the exposure-response relationship.

Despite these limitations, the findings of this study still provide several implications for occupational health practice. First, incorporation of cognitive function screening, such as MoCA, into occupational health surveillance programs for high-risk noise-exposed workers is recommended (32). Second, hearing protection is not only the core intervention for prevention of occupational noise-induced hearing loss, but may also provide additional benefits for preservation of long-term cognitive health in workers. Third, hypertension, as a modifiable cardiovascular risk factor, should be a key target of health promotion in occupational populations.

In conclusion, this study showed that high-frequency hearing loss in occupational noise-exposed populations was significantly and independently associated with cognitive decline, and that hearing loss was a key mediator in the relationship between noise exposure duration and cognitive function. Hypertension was another independent risk factor for cognitive impairment in this population. These findings support the integration of cognitive function assessment into occupational health surveillance systems and underscore the synergistic value of combining hearing protection with cardiovascular risk factor management to preserve cognitive health in occupational populations.

## Data Availability

The original contributions presented in the study are included in the article/supplementary material, further inquiries can be directed to the corresponding author.
