# Correction: Association between occupational noise-induced hearing loss and cognitive function: a multimodal cross-sectional study

**DOI:** 10.3389/fpubh.2026.1911313

**Published:** 2026-07-07

**Authors:** Qi Jin, Yangfu Bian, Cailing Zhou

**Affiliations:** Department of Occupational Disease, Hangzhou Hospital for the Prevention and Treatment of Occupational Disease, Hangzhou, China

**Keywords:** auditory brainstem response, cognitive impairment, hearing loss, hypertension, occupational noise exposure, smoking

The approval number in the **Ethics statement** was erroneously given as [HZF-IRB-2022-001]. The correct approval number in the **Ethics statement** is [杭职防伦审2025研第001号].

The original version of this article has been updated.

